# Bollingbroke House Pay Hospital

**Published:** 1892-12-03

**Authors:** 


### The Institutional Workshop.

### WITHIN THE HOSPITALS.

### BOLINGBROKE HOUSE PAY HOSPITAL.

An old-fashion suburban mansion, surrounded by just enough of its own turf and trees to keep it apart from the rest of the world, Bolingbroke House stands on the verge of Wandsworth Common. It has been a good house in its day—one of five, of sufficient importance to have given the country lane in which they stood the name of "Five House Lane," which of late years has given place to "Bolingbroke Grove." The other four have gone the way of many a fine old homestead in the rural environs of London; but Bolingbroke House was a few years ago saved from impending demolition by the Rev. Canon Erskine Clarke, who bought it at a cost altogether of about £6,000, and started it on a new career of usefulness as a "home in sickness."

There is no denying that a "home" in sickness sounds a good deal pleasanter than a "hospital" in sickness. One hasn't of course, a word to say in disparagement of the great London hospitals. No man of sense or right feeling can go through their great wards full of sufferers, surrounded by every comfort, and tended with the utmost possible care and skill, without fervently thanking God for them. But still they are great hospitals and not homes, and their wards are full of people who are strangers to each other, and many of whom belong to the roughest and most uncivilised of our working population. beautiful as our general hospitals are in their order, their cleanliness, their science, and management, it is not surprising that many sensitive people, when overtaken by sickness or by accident, feel that they would almost rather die than go into one of them. They shrink from the idea of public charity; accustomed to the privacy of their own bed-rooms, they dislike the semi-publicity of the bed in line with scores of others; they have the strongest aversion to anything in the nature of a clinical lecture over them; they cannot bear to be carried off from friends and relatives, and the idea of subjection to rigid rules and regulations, however essential to the efficient working of a great institution, is exceedingly repugnant to them.

Well, here is Bolingbroke House—just the place for such people—a great, old-fashioned mansion, spacious and light and airy, overlooking a breezy, sunlit common, and pervaded everywhere by women, gentle of face and quiet of speech, deft of hand, and full of genial kindness. The great windows of the home let in floods of light, the warming and ventilating seem perfect, and the whole interior is furnished sufficiently, though plainly, and fitted up with everything essential to the proper surgical and medical treatment of the sick inmates.

It is only a small hospital. They have conveniences for five-and-twenty or from that to thirty patients at a time, and during last year—their busiest year—they had only a hundred and forty-eight patients. But the smallness of the numbers of course renders it easier to deal with them as individuals, and to dispense to a great extent with any very Draconian system of rules and regulations. Individual preferences and inclinations may be considered—in matters not interfering in any way with medical treatment of course—to an extent that would be impracticable in a great general hospital, and in many little ways the endeavour to make the place a "home of sickness" is evidently very successful.

The original idea of the Bolingbroke was that it should be a place of kindly care and competent medical treatment for those who could pay for what they had. Not half enough thought and sympathy and help are expended on the sensitive, high-spirited, independent, and respectable poor, who sometimes in the race of life get thrown heavily. Sickness and accident often come disastrously enough to those who are in the midst of friends and in homes of comfort and convenience. To those who haven't friends and are living perhaps in lodgings or in houes too confined to offer any facilities for the proper treatment of the sick, illness or accident bring with them perplexities and troubles which greatly aggravate the misfortune, even though funds may not be altogether wanting. The main object of the Bolingbroke is to provide hospital accommodation for just such people. They are expected to pay not less than half a guinea a week, while those who can afford to pay three, four, or five guineas are required to do so. It has been found by practical experience that the benefits of the institution cost upon the average about a couple of guineas a week. The inmates all get whatever their circumstances require in the way of food, medical attendance, and nursing—excepting, it may be, some special operation which may have to be specially arranged for—but payment is to be made according to circumstances, the profit on one patient compensating for the loss on another. The hospital has been going since 1880, and at first it seemed that this idea could have been successfully carried out, and that the scheme would be entirely self-supporting. After a while, however, the notion of paying hospitals was very widely adopted, and so many competing institutions were set up for the reception of those who needed hospital treatment and could afford to pay well, that the Bolingbroke was left mainly with those who could pay very little. The purpose of the establishment is one of benevolence in the main, and it has been found impossible to avoid rather an overwhelming majority of the feebler folk whose fees can only partially cover their cost. They get here members of various professions, clerks, governesses, servants of a superior class, a few tradespeople, some who are dependent on friends who are unable to take them into their own homes, but who are willing to pay a little for them. A governess will come with a very slender purse. She has few friends, and no home to go to; but out of her scanty earnings she has managed to save a ten-pound note. Some tumour is developing and requires to be removed, and she may need not only expensive surgical skill, but weeks of nourishing diet and skilful nursing. She has a mortal horror of the hospital, but she cannot afford private treatment, and even if she could, she has no home to go to. Will they take her in? Yes; the committee will receive her, though it may be quite impossible to say how long it will be before she is off their hands. The committee take all her circumstances into account, and fix on a weekly fee that seems to be fair and reasonable, and she is tended with every care and kindness whether her case proves long or short. Another applicant for the hospitality of Bolingbroke House may be a young servant without home or friends. She has saved a five-pound note or so, and she is received for treatment in a similar way, though her scanty funds cannot be expected to pay anything like the cost of what she will receive. In the course of last year there were eighty surgical cases received, of whom sixty-five underwent operations, some of them of a very severe character. In no less than fifteen cases poor sufferers were received here with cancer of the breast, and were successfully operated on. What a haven it must be to a woman who is suffering and comparatively friendless, and perhaps may have been delicately nurtured, to find here a "home in sickness!"

It is just because they are sensitive and independent and self-respecting that many of them come here, and for that very reason all who can help such an institution ought to be eager to do so. Many of the inmates here, however, are by no means homeless or friendless, but they need surgical and medical attention and skilled nursing, which may be impracticable in their own homes, and therefore they come here. If they please they may have their own doctors and their own private rooms. Their families are relieved of the burden of a sick inmate, and they themselves will have the inestimable advantage of quiet and the best possible conditions for speedy recovery. There is a resident medical superintendent to see that the doctors' orders are duly carried out; there is thoroughly competent and skilful nursing, and all the conveniences and appliances of a well-appointed hospital are at their service.

It seems a thousand pities that such an institution should be in any degree crippled for want of funds. Yet such is the case. More than once in its brief career it has come to the verge of failure, and would certainly long ago have had to close its doors but for the noble liberality and perseverance of the Rev. Canon Clarke, the Vicar of Battersea. It is indebted also to many medical men of distinction. Nobody knows better than an experienced doctor the great need that exists for such hospitals, and some years ago a number of well-known hospital practitioners investigated the affairs of the institution, and, finding that under the benevolent vicar's control everything was thoroughly well managed, they constituted themselves honorary medical officers, and one of them, Mr. Thomas Bryant, F.R.C.S., M.Ch., undertook to become surgeon to the hospital. These gentlemen have greatly assisted the Bolingbroke by introducing a good many of the better paying class of patients, and receipts the last two or three years have gone up considerably. There is still, unfortunately, a difference of six or seven hundred pounds between receipts and expenditure, and it is on the wrong side, although there has been a remarkable amount of self-sacrificing devotion. The able young Medical Superintendent (Dr. Lyster) has given seven years of his life to promoting the interests of the Bolingbroke on merely nominal terms of remuneration; Mr. Bryant, too, seems to have given quite gratuitously the best of his great skill as a surgeon, and, says Canon Clarke, "has been as interested in them as if they had been his own private patients." An institution for the sick and suffering calling forth so noble a devotion ought to receive very liberal support, and it undoubtedly would do so if it were more widely known. Indeed, it is difficult to doubt that if Londoners generally could know the good that a place of this kind is calculated to do, there would soon be one in every district of London. It may be useful to say that, of 148 patients received last year, there were: Professionals, 32; clerks, 16; governesses, 10; tradespeople. 13; independent, 9; dependent on friends, 9; servants, 21; married women, 25; widows, 5; children, 8.

